# GATA-dependent transcriptional and epigenetic control of cardiac lineage specification and differentiation

**DOI:** 10.1007/s00018-015-1974-3

**Published:** 2015-07-01

**Authors:** Sonia Stefanovic, Vincent M. Christoffels

**Affiliations:** Department of Anatomy, Embryology and Physiology, Academic Medical Center, University of Amsterdam, Meibergdreef 15, 1105 AZ Amsterdam, The Netherlands

**Keywords:** Heart development, Cell fate decisions, Histone-modifying enzymes, GATA transcription factor, Chambers, Conduction system

## Abstract

Heart progenitor cells differentiate into various cell types including pacemaker and working cardiomyocytes. Cell-type specific gene expression is achieved by combinatorial interactions between tissue-specific transcription factors (TFs), co-factors, and chromatin remodelers and DNA binding elements in regulatory regions. Dysfunction of these transcriptional networks may result in congenital heart defects. Functional analysis of the regulatory DNA sequences has contributed substantially to the identification of the transcriptional network components and combinatorial interactions regulating the tissue-specific gene programs. GATA TFs have been identified as central players in these networks. In particular, GATA binding elements have emerged as a platform to recruit broadly active histone modification enzymes and cell-type-specific co-factors to drive cell-type-specific gene programs. Here, we discuss the role of GATA factors in cell fate decisions and differentiation in the developing heart.

## Introduction

Congenital heart defects affect nearly one percent of live births and are found in up to one-tenth of spontaneously aborted fetuses [1, 2]. The development of the heart is controlled by evolutionary conserved transcriptional networks. Genetic studies have identified mutations in genes encoding transcription factors as well as proteins organizing the chromatin structure that are responsible for congenital heart defects [3–5]. Defining the transcriptional networks underlying normal heart development is a prerequisite for understanding the molecular basis of congenital heart malformation. The specification of multipotent heart progenitor cells and their differentiation into different cell lineages is under tight spatial and temporal transcriptional control. Regulatory DNA elements (promoters, enhancers, repressors, and insulators/boundary elements) can interact with and respond to activating or repressing transcriptional complexes (transcription factors, activators, coactivators, repressors, and corepressors) to mediate the temporal and spatial control of transcription. Hence, functional analysis of DNA elements conferring cell-type-specific gene expression is a good starting point to identify the molecular mechanisms that underlie these patterns of gene expression. Dissection and analysis of regulatory DNA elements have led to the identification of many key regulators of the cardiac gene regulatory programs including the zinc finger TF Gata4. Mutations in both *GATA4* and its family member *GATA6* in patients have been associated with arrhythmias and defects in atrial, ventricular, and AV septation [6–11]. In this review, we will discuss the role of the GATA family of transcription factors in heart gene regulation and differentiation.

## Development of the chambers and the conduction system

**Fig. 1:**
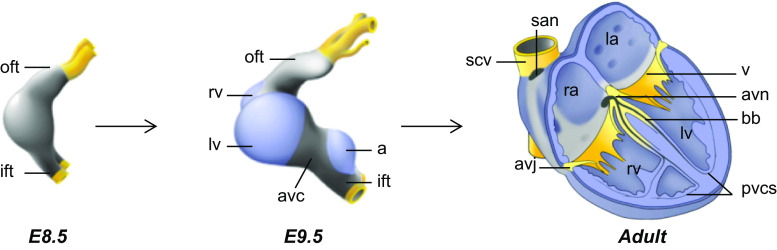
Schematic overview of heart development. The early heart tube has an embryonic phenotype (*gray*). Chamber myocardium (*blue*) expands from the outer curvatures, whereas non-chamber myocardium (*gray*) of the inflow tract, atrioventricular canal, outflow tract, and inner curvatures does not expand. *a* atrium, *bb* bundle branch, *ift* inflow tract, *la* left atrium, *lv* left ventricle, *pvcs* peripheral ventricular conduction system, *ra* right atrium, *rv* right ventricle, *scv* superior caval vein

In vertebrates, the heart arises from cells in the anterior lateral plate mesoderm of the early embryo (stage E7–7.5 in mouse). By E7.5–8.0, during folding of the embryo and formation of the foregut, these cells migrate to the midline to form the primary heart tube. The embryonic myocardium of the tube is characterized by a primitive phenotype, i.e., limited cell proliferation, a poorly developed contractile apparatus, and slow conduction [12, 13]. During further development, the heart tube elongates by addition of progenitor cells to its poles. These cells are referred to as second heart field progenitors that are kept in an undifferentiated and rapidly proliferating state dorsal to the primitive heart tube [14, 15]. Simultaneously, specific regions in the embryonic heart tube acquire a chamber-specific gene program, differentiate further, and expand by rapid proliferation to form the ventricular and atrial chamber myocardium (>E8.5). The chamber myocardium acquires properties of fast conduction, and the contractile apparatus starts to develop. In contrast, the regions flanking these differentiating chambers, the sinus venosus, the atrioventricular (AV) canal, and the outflow tract do not differentiate or expand and consequently form constrictions. The AV canal preserves characteristics of the embryonic myocardium of the tubular heart (Fig. 1), including its slow conductive property, ensuring AV delay of the impulse underlying the alternating contraction of the atria and ventricles. Signals from the AV canal are crucial for septation and valve formation, whereas the AV myocardium itself forms the AV node and AV ring bundles of the conduction system [16–18]. The fetal conduction system can be subdivided into more compartments such as the sinus node, the AV node, AV bundle, the left and right bundle branches, and the peripheral ventricular system. Each of these structures contains cardiomyocytes with pacemaker activity and specific properties that discriminate them from atrial and ventricular chamber myocardium.

Embryonic myocardial cells are confronted with a critical decision: differentiation into pacemaker-like cardiomyocytes of the AV canal or into working cardiomyocytes of the atrial and ventricular chambers. Bone morphogenetic protein 2 (*Bmp2*) is expressed in the primary heart tube where it stimulates the expression of T-box 2 (*Tbx2*), required for the development of the AV canal [17, 19, 20]. Both AV canal and chamber myocardium are competent to respond to BMP signaling [21, 22]. However, *Bmp2* is selectively expressed in the AV canal myocardium precursors and conditional deletion of the BMP type I receptor ALK3 in the conduction system using specific Cre driver mice (*cGata6*-Cre) demonstrated that BMP signaling regulates the proper development of the AV conduction system [23, 24]. Consistently, although the downstream effector of BMP2, Smad4, is ubiquitously expressed at E10.5, its localization (and transcriptional activity) is predominantly nuclear in the AV canal [25, 26]. Interestingly, insertion of a transgenic construct harboring the AV canal-specific enhancer of the chicken *Gata6* (*cGata6*) in the mouse genome leads to transgene activity in the cardiac crescent (E7.5) where both *Bmp2* and *Tbx2* are expressed [27, 28]. These observations, together with functional and lineage tracing analyses for *Tbx2,* support the notion that the AV canal myocardial cells are already specified at this early stage [18, 29, 30].

More broadly expressed cardiac transcription factors such as Tbx5, Tbx20, and Nkx2.5 are also required for the development of the conduction system [31, 32]. Chamber differentiation is suppressed by Tbx2 and Tbx3, which are selectively active in the AV canal myocardium where they suppress chamber-specific genes including *Nppa* (Anf, atrial natriuretic factor), *Gja1* (Cx43, connexin 43), and *Gja5* (Cx40, connexin 40) [33–35]. The sharp boundaries between these two compartments are obtained by repressive activities mediated by the Notch target genes *Hey1* and -*2* [17, 36]. *Hey1* is expressed in the atria and *Hey2* in the ventricles, but neither gene is found to be expressed in the AV canal myocardium [36, 37].

## Transcriptional activities of GATA factors in mammalian heart development

The family of GATA transcription factors consists of six members, Gata1-6. In vertebrates, most tissues of either mesodermal or endodermal origin express one or more Gata4-6 factor during development. In the primitive streak, *Gata4,* -*5*, and -*6* are detected in the precardiac mesoderm (stage E7.5) [38, 39]. The expression patterns of *Gata4* and -*6* have been reported to be identical throughout the primary heart tube (E8.0–8.5). As the tube loops (E8.5), *Gata5* becomes restricted to the endocardium, while *Gata4* and -*6* are maintained in the myocardium. *Gata4* is detected in the endocardium of the atria and ventricles and endocardial cushions of the AV canal and outflow tract. *Gata4* and -*6* are expressed in both the AV canal and chamber myocardium, with no evidence for differential expression levels and subcellular compartmentalization [38, 40–43]. Since the spatial organization of the nucleus may impact on activation or repression of gene expression and interaction with co-factors, assessment of the localization of GATA factors within the nucleus might be relevant.

Mice null for *Gata4* die between E8 and 9 because of defects in heart morphogenesis and ventral closure of the foregut. Aberrant heart formation in these mice is mainly secondary to defects in the extra-embryonic and definitive endoderm that affects migration and folding morphogenesis of the precardiogenic splanchnic mesodermal cells [44–46]. Using a tetraploid embryo complementation strategy, it has been possible to show that Gata4 is cell autonomously required for cardiogenesis [47]. Elegant functional analyses utilizing knockout ES cells and tissue-specific disruption in mice unequivocally established a role for Gata4, -5, and -6 in controlling heart development that is consistent with their expression pattern. Studies using heterozygous *Gata4* and *Gata6* mutants demonstrated that both factors are critical for general cellular processes including cardiac cell commitment, differentiation, proliferation, and survival, hampering the analysis of their function in the formation of structures that arise later [9, 11, 48]. The levels of Gata4 and -6 are critical for normal embryonic development and survival [9, 11, 48]. Embryos in which the expression of Gata4 protein in the myocardium is reduced by 50 % survive. In contrast, a reduction of 70 % causes fetal death between E13.5 and 16.5. These fetuses have AV canal defects, double outlet right ventricle, and hypoplastic ventricular myocardium. *GATA6* mutations cause persistent truncus arteriosus in human [49]. Gata6 also regulates morphogenetic patterning of the cardiac aortic arch [50]. Wnt2, a ligand expressed specifically in the cardiac inflow tract, acts with Gata6 to regulate the development of the atrial myocardium, pulmonary veins, and AV canal [51]. *Gata6* is downregulated in *Gata4* null mice and *Gata6* null embryos show down regulation of *Gata4*, indicating that these factors cross regulate each other [44]. In addition, *Gata6* compensates the loss of *Gata4* in inducing the myocardial gene program [39, 48]. This transcriptional interconnection is consistent with the observation that *Gata4* and *Gata6* double heterozygote mice die very early during embryonic development [48]. *Gata4* and *Gata6* mutants show defects of the venous pole region, suggesting involvement of these factors in embryonic inflow tract development and the venous blood connection [52]. Gata5 interacts with Gata4 and -6 in endocardial cushion formation, i.e., morphogenesis of the atrioventricular valves and outflow tract [53].

Gata4 is known to activate many cardiac genes displaying regionally restricted expression, including the chamber-specific genes *Nppa* and *Nppb* (Bnf, brain natriuretic factor) [54] and the contractile genes *Myh6* (α-MHC) and *Myh7* (β-MHC) [55, 56]. Gata4 is shown to activate the gene *Gja5* (Cx40) [57] that plays an important role in cell–cell communication in cardiomyocytes of the atrial chambers and in the ventricular conduction system [58]. Gata6 activates the ion exchanger gene *Ncx1* [59] and the potassium channel gene *Kv4.2* [60]. Expression of *Nkx2.5*, one of the earliest markers of the cardiac lineage in vertebrates, depends on GATA binding sites [61]. GATA factors also control the expression of *Mefc2c* in outflow tract and right ventricular myocardium [62]. Gata4 is a direct transcriptional activator of cell cycle genes *cyclin D2* and *Cdk4*. Gata4 physically and functionally interacts with CDK4 [63] and is required for cardiomyocyte proliferation [64]. Gata4 chromatin occupancy changes markedly between fetal and adult heart, with only partial overlap in binding sites [65]. This underlies the importance of rating the quality of ChIP-seq experiments and the datasets generated in order to preclude incomparability due to low quality.

## Interplay between GATA factors and chromatin

Approximately seven million GATA motifs are present in the human genome [11]. This raises the question of which motifs are occupied by Gata4 in vivo. With the development of ChIP-seq assays to assess Gata4 chromatin occupancy genome-wide, it has become clear that only a small subset of the GATA motifs are occupied in heart tissue, suggesting the existence of a GATA recognition code that dictates site occupancy [66–68]. How GATA factors discriminate among these motifs in a time- and location-dependent manner is unclear [69]. This question directly applies to Gata4, and -6 in heart development. An important consideration is whether Gata4 and Gata6 occupy identical or different WGATAR motifs in different myocardial compartments. Since Gata4 and -6 have redundant functions in regulating myocardial genes, addressing these questions might well explain their possible similar participation in the conduction system and chamber gene programs. Local chromatin environment, nearest neighboring factor binding motifs and intrinsic features of the WGATAR motifs are likely important parameters underlying the GATA recognition code. The transcriptional activation domains of Gata4, -5, and -6 are partially conserved, suggesting a similar mechanism of transcriptional activation within this Gata subfamily. Although Gata4, -5, and -6 bind a GATA or GATA-like sequence element, their individual affinities for various regulatory elements might also depend on flanking nucleotide sequences or on interactions with co-factors and other transcription factors. Gata4 ChIP-seq analyses also revealed that a large fraction of genomic regions occupied by Gata4 are not associated with a recognizable GATA binding site, indicating indirect binding [66–68]. Consistent with this, in vitro reporter assays suggest that Gata4 transcriptional activities do not necessarily require Gata4 DNA binding [11]. Since ChIP assesses protein-DNA proximity by cross linking and not by direct binding, it would be interesting to verify GATA binding using in vivo foot printing. Assessment of the genomic distribution of Gata4 and Gata6 occupancy by cell sorted-based genome and proteome-wide technologies should facilitate our understanding of how GATA factors select chromatin target sites. This has become a realistic option with the development of protocols to perform genome-wide analysis on small amounts of tissue [70–72].

There is no doubt that emerging molecular technologies will help in understanding the function of GATA TFs in cardiac lineage specification. GATA TFs regulate transcription during differentiation by mediating long-range DNA looping. Whether this is the case in the context of heart development is unknown. Chromosome conformation capture technologies (e.g., 3-5C, Hi-C) were developed to identify long-range chromatin interactions [73]. The ENCODE project has provided access to valuable data on genome-wide chromatin occupancy of transcription factors, chromatin-modifying and -remodeling enzymes, and histone modifications in heart tissues [74]. Merging these datasets can further facilitate identification of GATA regulatory elements. The functional importance of GATA elements can be assessed in vivo using recent genome editing technologies [75]. Recent ChIP-seq analyses show that in the context of cardiac stress, Gata4 binds to a subset of fetal genes such as *Nppb* [76] leading to the reactivation of the “fetal gene program.” However, reactivation of fetal genes constitutes a small portion of the transcriptional response of the diseased adult heart [65]. ChIP-seq and motif analysis have shown that the GATA motif is highly overrepresented in Gata4-bound regions from fetal, adult, and disease heart [65]. These analyses also indicate that the selection of GATA binding sites is influenced by the interaction of Gata4 with other TFs. The co-enriched motifs of other TFs are rather distinct between fetal-specific and adult-specific Gata4-bound regions. These changes are associated with altered expression of transcriptional regulators between fetal and adult heart. Interestingly, in hypertrophic hearts, Gata4 regions contain an atypical GATA motif and are enriched for the NFAT binding motif. NFATs are essential mediators of cardiac hypertrophy.

From studies in cell culture, two distinct DNA binding modes of GATA transcription factors have been deduced [77]. In the wrapping mode, the two zinc fingers of a GATA factor wrap around a palindromic GATA site, cooperatively enhancing binding affinity and kinetic stability. In the bridging mode, a single GATA DNA binding domain bridges two pieces of DNA. Because the DNA binding domains of GATA proteins are highly conserved, these structural features could be shared by all six GATA family members. This may have important implications for transcriptional regulation by GATA proteins. For example, varying concentrations of GATA proteins during heart development may not just affect their occupancy of DNA but also could switch their DNA binding mode and affect transcriptional networks. GATA proteins may loop DNA through co-factors (e.g., Fog proteins) [78], which have also been shown to play important roles in mediating DNA loop formation [79–81].

Mechanisms underlying the function of governing the decision of whether GATA TFs function as activators or repressors of a targets gene have been studied in depth in the context of the hematopoietic system. Using the aforementioned approaches, it has been shown that in this lineage context, Gata1 uses Fog1 to activate and or repress the majority of its target genes [82, 83]. Fog1 associates with the repressive nucleosome remodeling and deacetylase NuRD complex [84]. The transcription factor Gata1 is required for terminal erythroid maturation and functions as an activator or repressor depending on gene context [85]. During late erythroid development, PRC2 complex is involved in late stages of silencing of some Gata1 repressed genes. The Gata1-interacting coregulator Friend of Gata-1 (Fog1) mediates Gata1-dependent activation and repression in a context-dependent manner [82, 83]. Fog1 facilitates Gata1 chromatin occupancy and interacts with the repressive nucleosome remodeling and deacetylase (NuRD) complex [84]. GATA TFs in the context of the hematopoietic system are known to cooperate with Brahma-related gene-1 (BRG1), an enzyme that disrupts chromatin structure, acting as either an activator or a repressor in the SWI-SNF nucleosome remodeling complex [86, 87]. BRG1 could be a privileged mediator or partner in the action of cardiac GATA factors on chromatin structure.

Chromatin remodeling enzymes regulate Gata4 target genes by post-translational modifications of Gata4 [11, 88]. Gata4 physically interacts with histone acetyl transferases (HATs) and histone deacetylases (HDACs) [89–91]. Activation of Gata4 is mediated through its acetylation by the HAT p300 [92] which is consistent with the large proportion of cardiac enhancers occupied by both [43, 65]. In turn, p300 also acetylates histones such as the histone 3 at lysine 27 (H3K27ac), a mark associated with active enhancers [93]. Gata4 cooperates with the acetylase p300 to deposit H3K27ac at cardiac enhancers to stimulate transcription [65]. Paradoxically, p300 occupies both GATA-activated and -repressed genes [94, 95]. A large portion of Gata4 occupancy occurs at sites that have a closed chromatin configuration in the normal heart [65]. HDAC1and HDAC2 can physically interact with Gata4 [89–91] and are functionally redundant in the heart [96]. Gata4 forms a complex with Hopx which serves as an adapter to facilitate HDAC2 recruitment, which in turn reduces Gata4 activity by deacetylation [88]. The activity of GATA factors can also be regulated through methylation. The polycomb-repressive complex 2 (PRC2) directly binds and methylates Gata4, reducing its interaction with and acetylation by the HAT p300 [97]. This results in attenuation of Gata4 transcriptional activity [97]. Given the interplay between GATA factors and multiple coregulators, a system biology approach will be required to develop a comprehensive model for the cardiac transcriptional networks around the GATA factors.

## GATA factors interact with other heart-expressed transcription factors

GATA factors have a dual finger module which is highly conserved among the six mammalian proteins. The C-terminal finger mediates sequence-specific DNA binding, while the N-terminal finger modulates DNA binding and contacts DNA. The zinc fingers interact with multiple coregulators. Disruption of these interactions has been reported to underlie congenital heart defects [6]. A precise balance between interactions with co-factors appears essential for the regulation of their spatial and temporal activity. It was previously demonstrated by in vitro and in vivo studies that Gata4 and Tbx5 physically interact and cooperatively activate endocardial [98] and myocardial genes such as *Nppa* [6]. Gata6 and Tbx5 genetically interact in vivo [40]. Combinatorial expression of Gata4, Nkx2.5, and SRF (serum response factor) directs early cardiac gene activity [66, 68, 99]. Although the Gata4 and Gata6 protein interaction with the primary myocardium/conduction system-specific protein Tbx3 has not been firmly established, ChIP-seq data for Gata4 and Tbx3 from heart tissue revealed a large portion of overlapping peaks, suggesting that Tbx3 and Gata4 might cooperatively repress the working myocardial gene program [67]. Gata6 regulates transcription of *Id2* [100], a transcriptional repressor involved in conduction system lineage specification [31]. Gata4 and Smad4 form protein–protein interactions and cooperatively regulate cardiac valve development by regulating *Id2* [101]. Further studies will reveal whether Gata4 also activates *Id2* in the conduction system and whether a Gata4/6-Smad4-Id2 network module participates in the normal control of conduction system gene expression and specification. Studies in postnatal cardiomyocytes demonstrated that Gata4 and Gata6 can functionally interact with each other, at a single GATA element, to synergistically activate the *Nppa* and *Nppb* promoters [102]. Retinoic acid receptors regulate *Nppa* and *Nppb* genes via direct interaction with Gata4 and its co-repressor, Fog2 [103]. Mutations of Gata4 in human and in mouse models can result in abrogation of physical interaction with some of these co-factors [104].

## GATA factors and conduction system-specific enhancers

**Fig. 2:**
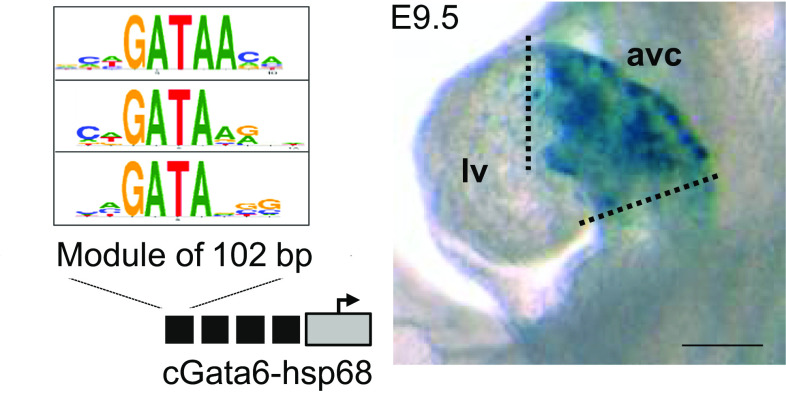
Tandemly repeated copies of a 102 bp cGata6 enhancer fragment drive expression in the developing AV canal at stage E9.5; a module of 102 bp contains 3 GATA binding sites. *Scale bars* 100 μm

DNA elements conferring cell-type-specific gene expression are ideal to analyze the molecular mechanisms that underlie the localized gene expression of conduction system genes. For example, one of the enhancers that flank the cGata6 gene directs transgene expression throughout the entire primary heart tube and in all regions of primary myocardium that persist at later stages (Fig. 2) [27, 43, 105]. Interestingly, this enhancer module is completely inactivated in newly arising chamber myocardium. Several other DNA elements conferring AV-restricted patterns of gene activity have been identified in mouse transgenic embryos, enhancers regulating *Tbx2* [106], *Tbx3* [107], *Gjd3* (Cx30.2, connexin 30.2) involved in conduction slowing [41], and the promoter of *cTnI* [108]. Interestingly, the common features of these AV canal-specific regulatory elements are the presence of GATA consensus sequences and in vivo Gata4 occupancy as demonstrated by recent ChIP studies [65, 67]. Moreover, mutations in Gata4 and -6 are responsible for congenital heart defects including AV canal defects and arrhythmias in human and mouse [8, 11, 41, 109], implicating their function in conduction system tissues [100, 110–113]. Conduction system-restricted gene activity is controlled by more broadly expressed transcription factors. We and others have shown that the activity of these AV canal-specific regulatory elements depend on GATA binding sites [41, 43, 105]. The activity of the *Gjd3*-AV canal enhancer also depends on the broadly expressed factor Tbx5 [41]. Gata4, Tbx5, and Nkx2.5 are expressed in largely overlapping domains in the conduction system and co-occupy several conduction system gene loci [67]. This suggests that they may cooperatively activate genes in the conduction system [114]. Despite widespread expression throughout the myocardium, these factors have a specific role in patterning the AV conduction system.

## How do GATA factors acquire their AV canal-specific functions?

The emergence of cell sorting-based transcriptomics and ChIP-seq is contributing to a better understanding on how GATA factors acquire specific functions. By integrating a variety of ChIP-seq datasets, we found that Gata4 binds to loci associated with both active and repressive chromatin signatures [43]. We tested whether the AV canal-specificity of these enhancers involves a mechanism of repression outside the AV canal. The promoter of the rat cardiac troponin T (cTnT, Tnnt2) gene drives efficient pan-cardiac expression in transgenic mice [115]. Previously identified AV canal enhancers were coupled to the *cTnT*-*lacZ* transgenic construct, and a repressive function was assayed in mouse transgenic embryos.

Analysis of ChIP-seq data from tissue-specific compartments coupled with validation in transgenics revealed these AV canal enhancers act as cell-type-specific switches, activating transcription in the AV canal and repressing transcription in the chambers. We found that mutation of the GATA binding sites affected the repressive activity of the *cGata6* and *Tbx2* enhancers. Thus, GATA-dependent AV canal-specific enhancers mediate both region-dependent gene activation and repression. Gata4 activates such enhancers in synergy with Bmp2/Smad signaling. Our data indicated that GATA sites within AV canal-specific regulatory regions recruit a Gata4-Smad4-HAT p300 transcriptional activation complex. This is associated with AV canal-specific acetylation of histone H3K27.

In chamber myocardium, Gata4 represses these enhancers through pan-cardiac HDACs and chamber-specific Notch target genes Hey1 and -2 [43]. The *cGata6*, *Gjd3*, and *cTnI* modules do not contain a consensus Hey binding site (i.e., E-box), suggesting that Hey factors mediate repression via GATA protein interaction [116]. We speculate that Gata4-HDAC mediated deacetylation of H3K27 serves as a mode of confinement of the conduction system gene program, preventing its activity in the chambers. Interestingly, histological analysis revealed that Tbx2 expression was only slightly expanded in Hey1 and Hey2 double-deficient mice [117]. This implies that Hey1 and -2 refine the boundary between the AV canal and the chambers by repressing *Tbx2* [36]. These factors contribute but are not essential for chamber repression by Gata4-HDACs.

**Fig. 3:**
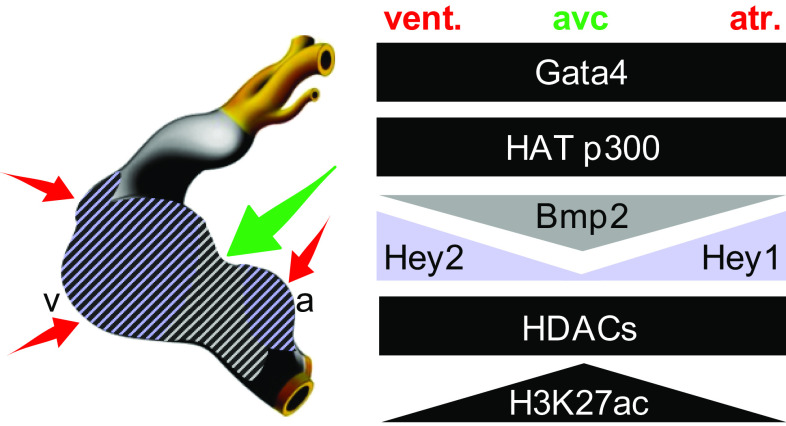
Cartoon depicting the overlapping expression patterns of Gata4, broadly active histone modification enzymes, localized co-factors, and the level of H3K27ac enrichment on AV canal gene loci at stage E9.5–E10.5, *atr* atria, *vent* ventricles

GATA sites in the AV canal regulatory elements thus serve as a platform to recruit broadly active histone modification enzymes and localized co-factors to drive AV canal-specific gene activity (Fig. 3). Obviously, post-translational modifications could play a role in this process such as direct acetylation/deacetylation of Gata4, -6 proteins accompanied by methylation by the aforementioned PRC2 complex. Whether a change of GATA factor concentrations guide this dual function is unknown. Since inspection of *Gata4*, *Bmp2,* and *cGata6*-lacZ expression patterns revealed that they are already expressed in the cardiac crescent, it suggests that already at the primitive streak stage, the GATA-Smad-HAT complex may occupy primary myocardial gene loci. This could lead to acetylation of H3K27 at this early stage. Subsequently upon chamber formation, these loci may become deacetylated in cells receiving appropriate local guidance cues.

## Perspectives on the requirement of GATA factors to reprogram pacemaker cell identity

Disease, aging, or gene defects may cause conduction system dysfunction, a common cardiac disorder that currently requires the implantation of an electronic pacemaker. Recent advances in the stem cell field indicate that the creation of induced pacemaker cells may offer a promising alternative to repair diseased pacemaker myocardium [118]. Developmental and genetic studies have identified several cardiac transcription factors that are necessary for the development of the conduction system [13]. Currently, there is interest in using this information to reprogram cells to pacemaker cells by transducing TF genes [119]. In vitro direct reprogramming of human fibroblasts toward a cardiomyocyte-like cell type requires a minimal cocktail of transcription factors that include Gata4 [120, 121]. However, in vitro reprogramming remains inefficient and better understanding of epigenetic changes associated with TF overexpression may provide clues for improving the efficiency [122]. A key question is how factors such as Gata4 could enable cellular reprogramming. Gata4 occupancy does not necessarily imply that it activates transcription. Nevertheless, its occupancy seems to initiate a series of changes that allow the enhancer to recruit its full set of transcription factors and chromatin remodeling complexes. At a particular stage in differentiation, some transcription factors, defined as "pioneer factors," are able to access DNA target sites in chromatin where other factors are not yet able [123]. These factors can recruit chromatin modifiers and activate or repress transcription. Similarly, Gata4 has been proposed to initiate formation of transcriptional regulatory complexes on closed chromatin [124]. Later during differentiation and depending on the context such as the presence of binding sites for other transcription factors, an enhancer may become associated with active histone marks such as the H3K27ac or with repressive marks and the poised RNA pol II complex. As development continues, we and others have shown that the binding of additional factors or changes in cofactor availability may change the activity of the targeted element. This change of activity occurs when the binding of the priming factor is maintained which enables a rapid transcriptional response to inductive signals. In the lineage conversion of one cell type to another, overexpression of GATA factors may actively open-up the local chromatin and make it competent for other factors to bind.

In many of the reprogramming studies, multiple factors are required for the cellular conversion, emphasizing the role of cooperativity among factors in the starting population of cells. Binding cooperatively could be sufficient to simultaneously engage a target site in chromatin and activate gene expression. Stoichiometry of Gata4 and its co-factors influences the efficiency and quality of cardiomyocyte reprogramming [125]. Although the methods using this cocktail of cardiac transcription factors provided the proof of concept that somatic cells can be reprogrammed into cardiomyocytes, reprogramming toward specific human cardiac subtypes is a remaining challenge. Moreover, only a small subset of the targeted cells becomes reprogrammed in vitro. Inhibitors of histone-modifying enzymes are known to enhance reprogramming, suggesting that modulation of these chromatin-modifying enzymes may be exploited to more efficiently generate induced pacemaker cells [126–128]. In this regard, it remains to be determined whether manipulating the GATA-Smad-HAT/GATA-Hey-HDAC complexes could target many silent conduction system-specific sites, open the chromatin for active transcription and enhance the reprogramming toward human pacemaker cells efficiently.

**Fig. 4:**
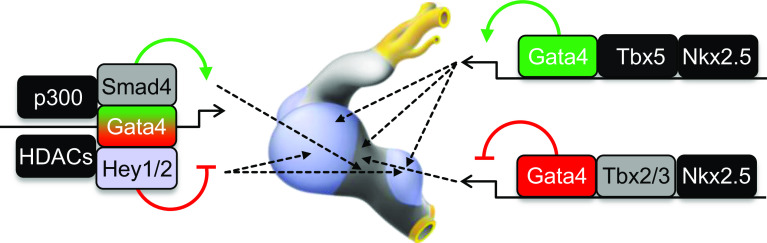
Working model of a GATA factor regulatory network for primary versus chamber myocardium formation. GATA elements direct complex formation of cardiac transcription factors, broadly active histone modification enzymes, and localized co-factors to drive specific gene activity. GATA and other broadly expressed transcription factors cooperatively activate chamber genes in the chambers and AV canal genes in the AV canal. Locally expressed Tbx2/3 and GATA factors cooperatively repress the working myocardial gene program in the AV canal/primary myocardium. GATA binding site enhancers recruit a Gata4/Smad4/HATp300 transcriptional activation complex in the AV canal and a Gata4/Hey1,2/HDACs transcriptional repression complex in the chambers, which coordinately establish AV canal-specific gene expression. Broadly expressed, primary- and chamber-specific transcription factors are depicted in *black*, *gray,* and *blue*, respectively

In summary, the cooperation of GATA factors with active histone modification enzymes and localized co-factors ensures proper spatial–temporal expression of cardiac genes during normal embryonic development (Fig. 4). Defining how GATA or GATA-co-factors act in different myocardial compartments to enable epigenetic regulatory events will provide insight into the biology of progenitor cells leading to methods for increasing the efficiency of directed differentiation of pluripotent cells and cellular reprogramming into myocardial subtypes. Addressing this question is also critical for understanding the origin of congenital heart defects.

## Abbreviations

ANF: Atrial natriuretic factor
AV: Atrioventricular
BMP: Bone morphogenetic peptide
BNF: Brain natriuretic factor
E: Embryonic day
HAT: Histone acetyl transferase
HDAC: Histone deacetylase
TBE: T-box element
TF: Transcription factor
